# Perceptual interpretation of biological motion relates to autistic traits in children born very preterm

**DOI:** 10.1007/s00221-025-07186-6

**Published:** 2025-11-04

**Authors:** Martin Johansson, Olga Kochukhova, Eva Larsson, Cecilia Montgomery, Ylva Fredriksson Kaul

**Affiliations:** 1 Department of Women’s and Children’s Health, Uppsala University Hospital, Uppsala University, 751 85 Uppsala, Sweden; 2 Uppsala University Children’s Hospital, Uppsala, Sweden; 3 Department of Psychology, Uppsala University, Uppsala, Sweden; 4 Department of Surgical Sciences / Ophthalmology, Uppsala University, Uppsala, Sweden; 5 Department of Surgical Sciences / Neuroradiology, Uppsala University, Uppsala, Sweden

**Keywords:** Autism, Biological motion, Extremely preterm, Neurodevelopment, Perception, Vision

## Abstract

Children born very preterm (VPT, ≥ 28 to < 32 gestational weeks) and extremely preterm (EPT, < 28 weeks) are at higher risk for autistic traits and biological motion (BM) processing deficits. Thus, we aimed to examine links between autistic traits and a condensed BM interpretation assessment, as well as potential group differences in performance in 12-year-old children born preterm and full-term. Four short BM stimuli (point-light-walkers) in two noise levels were presented to 25 EPT, 53 VPT and 48 full-term 12-year-old children. Accuracy in BM interpretation was compared across groups and analyzed with parental ratings on the Social Responsiveness Scale 2, adjusting for neonatal characteristics and intelligence. Interactions between preterm status and BM interpretation accuracy with autistic traits were explored. Results showed that the children born EPT had poorer accuracy interpreting BM than the other groups, and children born VPT showed poorer accuracy BM interpretation in high noise compared with the full-term group. BM interpretation accuracy was linked to autistic traits in the EPT and VPT groups. Children born preterm with the poorest BM interpretation accuracy also exhibited the most autistic traits. We concluded that the condensed assessment found prematurity-related deficits in BM interpretation. Performance was strongly linked to autistic traits in the preterm groups, highlighting the relevance of BM interpretation for social reciprocity in children born VPT and EPT.

**Supplementary Information:**

The online version contains supplementary material available at 10.1007/s00221-025-07186-6.

## Introduction

Children born below 32 weeks of gestational age (GA) are at risk of deficits in visual perceptual abilities, social functioning and higher incidence of autistic traits, with the highest incidence observed in extremely preterm born children (GA < 28 weeks) (Allotey et al. 2018; Atkinson and Braddick 2007; Ritchie et al. 2015). Male sex, lower gestational age and deficits in general intelligence have been identified as risk factors for autism spectrum disorder (ASD) and autistic traits in children born very preterm (Laverty et al. 2021). Moreover, neonatal brain injury such as interventricular hemorrhage has been associated with later autistic traits (Kuzniewicz et al. 2014). While perinatal characteristics and neonatal brain injury are linked to developmental outcomes, they do not fully explain the differences in individual developmental paths (Allotey et al. 2018; Laverty et al. 2021). Autistic traits, such as subtle social communication difficulties, restricted interests, or sensory sensitivities, are also more common in children born very preterm and may only become evident as children grow older and face increasing social demands (Laverty et al. 2021; Vlaeminck et al. 2020). Previous research has demonstrated a high prevalence of ASD and pronounced autistic traits among children born extremely preterm in early adolescence (Laverty et al. 2021; O’Reilly et al. 2021). While ASD is most often clinically assessed during preschool and early school years, our focus was on the broader spectrum of autistic traits, including more subtle difficulties that are more likely to become apparent by adolescence. Even when these traits do not meet diagnostic criteria for ASD, they can still affect daily life over the lifespan. Visual perceptual ability, particularly social perception, has been linked to severity of autistic traits in children with ASD regardless of gestational age (Atkinson 2009; Federici et al. 2020; Foglia et al. 2022).

Visual perception and processing difficulties are also often displayed by children born very preterm and include problems with spatial awareness, motion perception, and interpretation of moving stimuli (Atkinson and Braddick 2007; Geldof et al. 2012; Kaul et al. 2016; Strand Brodd et al. 2012). In particular, social functioning and autistic traits have been linked to the processing of biological motion (BM), which refers to the movement patterns of living beings (Pavlova 2012). Most studies use human figure motion as stimuli, focusing on detecting BM and interpreting actions to infer emotions and intentions (Federici et al. 2020; Miller and Saygin 2013; Pavlova 2012). Biological motion processing is studied using point-light displays, where dots of light form a moving human figure (Cutting 1978; Pavlova 2012). Biological Motion stimuli are often embedded in visual noise-randomly moving dots that increase ambiguity (Cutting et al. 1988; Hadad et al. 2011; Pavlova et al. 2001). Existing literature has shown that at five years of age, typically developing children show adult proficiency in determining actions from BM presented without noise. Therefore, studies in older children and adults typically add visual noise (Pavlova et al. 2001). Detection sensitivity is measured by the highest noise level at which BM remains detectable. Children born very preterm have shown reduced BM detection sensitivity and difficulty interpreting BM at ages 5–17 years (Pavlova et al. 2005; Taylor et al. 2009; Williamson et al. 2015). Interpreting BM, especially with added noise, was also related to autistic traits in a study of 8–11 year old children born preterm. In the current study, we examined children born very preterm as they entered early adolescence (around age 12), a stage when social-cognitive demands increase and difficulties may become more apparent. Whereas earlier investigations relied on extensive assessments, conducting a shorter evaluation might provide an opportunity to examine the robustness of the previously shown associations between autistic traits and BM interpretation.

The primary aim of this study was to explore the associations between autistic traits and BM interpretation in a condensed assessment in 12-year-old children born very (GA 28 to < 32 weeks)- and extremely (GA < 28 weeks) preterm and full-term controls. To this end, secondary aims were to investigate potential group differences in BM interpretation accuracy and prevalence of autistic traits. Additionally, we aimed to determine the influence of covariates, including neonatal factors, concurrent ophthalmological factors and general intelligence, in order to isolate the BM interpretation accuracy’s connection to autistic traits.

## Methods

### Participants

The LOVIS project is a prospective study of a regional cohort of 113 very preterm children born in Uppsala County between 2004–2007 of whom 109 survived the first year (Strand Brodd et al. 2012). Perinatal and neonatal characteristics, including GA, sex, birth weight, small for gestational age status (birth weight below -2 standard deviations of the mean gestational age specific birth weight as defined by Marsál et al. (1996), administration of antenatal steroids, and presence of neonatal complications, were collected. Neonatal characteristics also included bronchopulmonary dysplasia, persistent ductus arteriosus, intraventricular hemorrhage (IVH) grade 3–4, cystic periventricular leukomalacia (PVL), and retinopathy of prematurity stage 3 or higher. No child had necrotizing enterocolitis, and none received postnatal steroids. Neonatal characteristics are important predictors of outcome after very preterm birth, and thus we investigated associations between neonatal characteristics and autistic traits as potential covariates. Seventy-eight (72%) children underwent assessment at 12 years. The children were divided into an extremely preterm subgroup (n = 25, referred to as the *EPT group,* GA 22–27 weeks) and a very preterm subgroup (n = 53, referred to as the *VPT group,* GA 28–31 weeks). As previously reported, the participating children had a slightly higher prevalence of PVL than the original cohort (Johansson et al. 2023; Kochukhova et al. 2022). Forty-eight children born at term were assessed as controls. Data collection was delayed due to the COVID-19 pandemic for 17 of these full-term controls. These children were about one year older at assessment but did not differ in outcome variables, compared with the first part of the control group.

Among the participants, six children, including one full-term control, had ASD based on clinical evaluation including the Autism Diagnostic Observation Schedule. The ASD diagnoses were established in the general health care prior to the 12-year follow-up.

All children’s legal guardians provided written informed consent, and the local ethics committee approved the study (Ups 03-665 and 2016/400).

### Procedure and materials

The assessments were conducted at the Uppsala University Children’s Hospital, Sweden. All children were assessed with best-corrected visual acuity.

#### Autistic traits

*Autistic traits* were the primary outcome and assessed using parental reports of the Social Responsiveness Scale, second edition (SRS-2) (Constantino 2012). The SRS-2 Total Score gave the overall level of autistic traits. Moreover, it gave specific subscale scores for Social Awareness, Social Cognition, Social Communication, Social Motivation, and Restrictive Repetitive Behaviors. All SRS-2 scores were standardized T-scores, m (SD) 50 (10), where scores ≥ 60 (1 SD above mean) were considered within clinical range of social- and reciprocity behaviors constituting autistic traits (Constantino 2012). The children diagnosed with ASD prior to the assessment were retained in the study group, as we were interested in autistic traits as a dimension covering the whole spectrum of difficulties.

#### Experimental set up

**Fig. 1:**
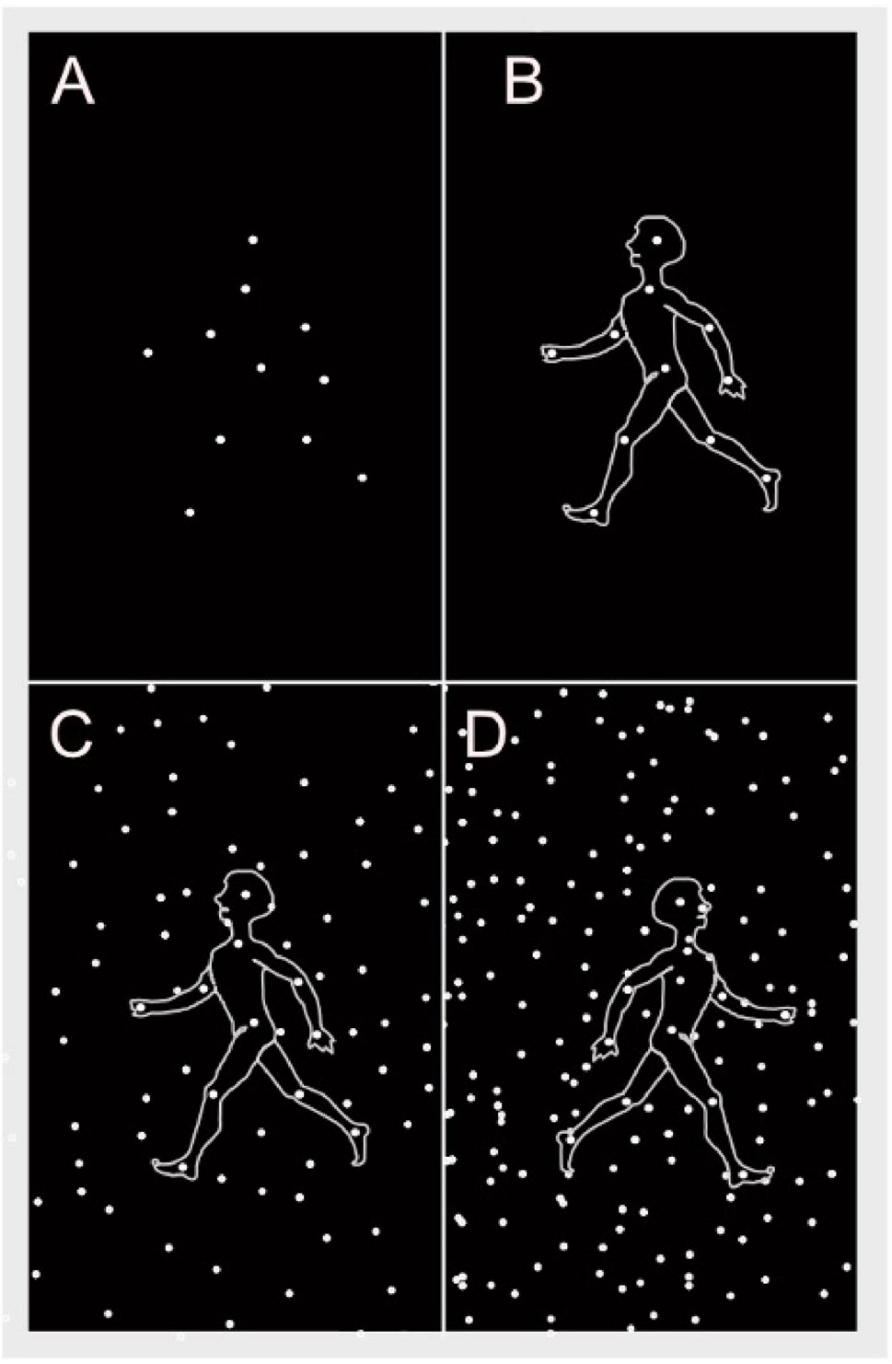
Static representation of the movie sequence used as biological motion stimuli point-light walker. Panel **A**: the placement of the dots on the joints of the body. Panel **B**: Outline of the body is shown for illustration, as the motion pattern of the dots enables perceiving a coherent human structure. Panel **C**: Biological motion in low noise. Panel **D**: Biological motion in high noise

The BM stimuli were presented on a 21-inch screen (41.28 × 22.70 visual degrees) with a resolution of 1500 × 844 pixels. A *familiarization trial* presented a side view of a point-light walker (size 3.1 × 6.2 visual degrees, 1 Hz step frequency) walking in place, masked by random dot motion. The walker was composed of eleven dots positioned at the head and major joints (shoulders, elbows, hands, knees, and feet) (Fig. 1). The mask started with 88 dots in 3.1 × 6.2 degrees, fading to 0 dots over 19 s, leaving only the point-light-walker visible for 5 s.

*In experimental trials***,** the same stimuli walking in place towards the left or right were used. In the low noise condition, the point-light walker was masked by 22 dots per 3.1 × 6.2 visual degrees (Fig. 1B), and in the high noise condition, by 44 dots per 3.1 × 6.2 visual degrees (Fig. 1C). The noise-dots had the same properties in terms of size and velocity as the dots in the biological motion pattern, however moving at random. The presentation time was adapted from Williamson et al., while noise levels were adapted from Williamson et al. (2015) as well as on Cutting et al. (1988); parameters were further refined through pilot testing. The children were asked to indicate whether each point-light walker was heading left or right. For brevity, four point-light walkers (two in each noise level) were presented, in randomized order for 0.5 s each. The children were instructed by text on the screen and in speakerphones that “now, the person is in the middle, in what direction is the person walking?”. Following each point-light-walker presentation participants were asked to identify “in what direction was the person walking?”. Participants answered verbally or by pointing. The BM stimuli were custom-made in JavaScript (Kaul A.) following Cutting (1978) and Cutting et al. (1988)

#### Additional measures

*General intelligence* was measured during the same day with the Wechsler Intelligence Scales for Children, fifth edition giving a Full-Scale IQ (WISC-V) (Wechsler 2014). We chose to use Full-Scale IQ as a covariate to exclude the potential effect of general intelligence on the autistic traits.

*Ophthalmological examinations* including visual acuity and low contrast visual acuity were conducted during the same day by an orthoptist/ophthalmologist after the neuropsychological assessment (very preterm group, n = 60, control group, n = 46) (Johansson et al. 2023; Karimi et al. 2023). Best-corrected visual acuity at distance (3 m) and near (40 cm) was measured binocularly with a logMar Sloan letters (Good-Lite) where visual acuity ≤ 0.8 Snellen decimal acuity were considered subnormal. Low contrast visual acuity was measured binocularly with the Lea Hyvärinen 2.5% low contrast chart (Good-Lite) and results below 0.5 was considered subnormal. Concurrent visual acuity at far and near, and low contrast visual acuity were included to account for variability in visual perception that could influence task performance.

### Statistical procedure

Data were assessed for homogeneity with Shapiro-Wilks test, whereafter both parametric and nonparametric tests were used as appropriate. Background variables were compared with t-tests or χ^2^.

For each level of noise (low and high), the results for BM interpretation accuracy were set on an ordinal level: both incorrect: 0, one incorrect: 1, both correct: 2. χ^2^ tests were used to examine potential group differences in BM interpretation accuracy by prematurity status (EPT, VPT, and full-term). To investigate within group difference in performance between the low and high noise Wilcoxon signed rank tests were used for each group.

As expected for the SRS-2, score distributions were positively skewed. To examine whether skewness differed between groups, we compared the SRS-2 Total score distributions using the Kolmogorov–Smirnov test, which indicated no significant group differences. Thus, while scores were skewed, the distributions appeared comparable across groups and did not bias the group comparisons or regression analyses.

One-way ANOVAs with Bonferroni correction were used to examine potential group differences in the sub domains of autistic traits by preterm-status (EPT, VPT and full-term).

For the primary aim, we explored associations between autistic traits as the dependent variables, and BM interpretation accuracy and background characteristics as independent factors with unadjusted linear regressions. Significant univariate associations between autistic traits and BM interpretation accuracy were further examined in multivariate linear regressions. Full-scale IQ was included as a potential covariate in all models, and, given the limited sample size in the EPT group, we adjusted for the neonatal characteristic most strongly related to the autistic traits. Regression coefficients < 0.2 were considered very weak, > 0.2 weak, > 0.4 moderate, > 0.6 strong and > 0.8 very strong (Campbell and Jacques 2023).

## Results

### Group differences in biological motion interpretation accuracy and autistic traits

#### Biological motion interpretation accuracy

**Fig. 2:**
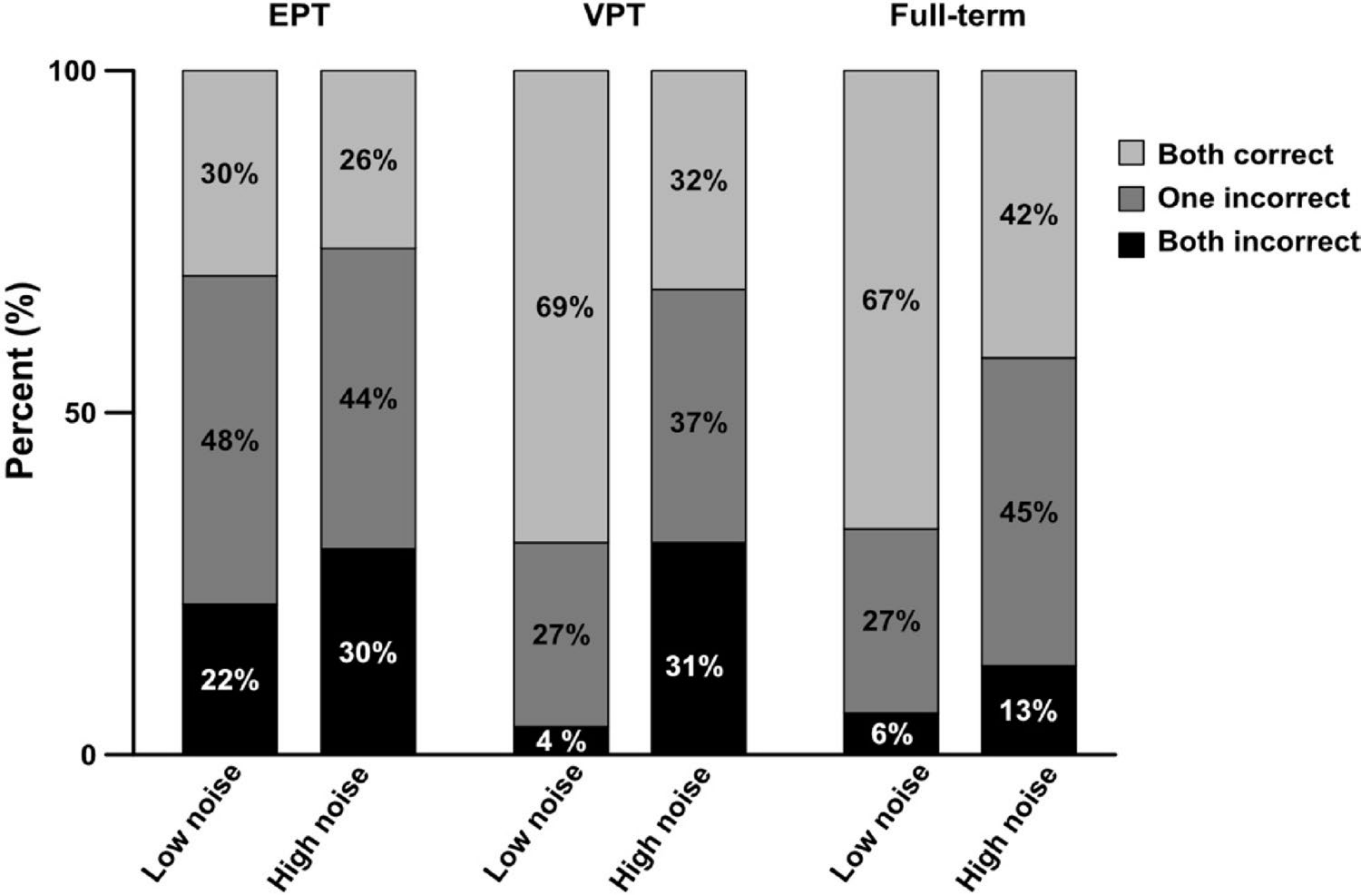
Depiction of categorized accuracy in interpreting biological motion. Results are presented for each gestational age group separately. The bars at the bottom (black) indicates proportion of children that could not interpret biological motion on any of the trials, the middle the proportion (grey) that correctly interpreted one, and the top (light grey) the proportion that correctly interpreted two

Descriptive statistics for background and outcome characteristics are found in Table 1. The categorized performances for BM interpretation accuracy (both incorrect, one incorrect, both correct) are displayed in Fig. 2. χ^2^ tests revealed group differences in performance in the low noise condition χ^2^(4, *N* = 120) = 13.29, *p* = 0.01), Cramer’s V = 0.23, indicating a medium effect. As seen in Fig. 2, the EPT group performed most poorly. In contrast, no significant association was found in the high noise condition χ^2^(4, *N* = 120) = 4.59, *p* = 0.33), Cramer’s V = 0.14 indicating a weak effect.

**Table 1:** Descriptive statistics

	LOVIS cohort	EPT group	VPT group	Full-term group
Neonatal characteristics	n = 78	n = 25	n = 53	n = 53
Gestational age, weeks	28.6 (2.3)	25.7 (1.5)	29.9 (1)	
Birth weight, grams	1198 (343)	858 (217)	1365 (259)	
Female, n (%)	35 (45)	12 (48)	23 (43)	20 (42)
Small for gestational age, n (%)	13 (17)	2 (8)	11 (21)	
Antenatal steroids, n (%)	56 (72)	18 (72)	38 (73)	
Bronchopulmonary dysplasia, n (%)	18 (23)	13 (52)	5 (9)	
Persistent ductus arteriosus, n (%)	19 (24)	13 (52)	6 (11)	
Intraventricular haemorrhage ≥ stage 3, n (%)	4 (5)	2 (8)	11 (21)	
Periventricular leukomalacia, n (%)	7 (9)	0	7 (13)	
Retinopathy of prematurity ≥ stage 3, n (%)	4 (5)	4 (16)	0	
12 year outcome				
Autism spectrum disorder, n (%)	5 (6.3)	2 (8)	3 (6)	1 (2)
Cerebral Palsy, n (%)	8 (10)	2 (8)	6 (12)	0
Age at assessment, years	12 (0.29)	11.9 (0.23)	12 (0.39)	12.4 (0.29)
Social responsiveness scale-2				
SRS-2 Total score	50.4 (11.2)	51.2 (11)	50.1 (11.3)	48.1 (9.5)
Range	39–90	39–78	39–90	38–82
Social awareness	48 (11.9)	49.7 (12.6)	47.2 (11.5)	47.3 (10.5)
Range	33–82	36–82	33–73	33–79
Social cognition	50.5 (11.9)	52 (12.6)	49.8 (11.6)	48.3 (9.7)
Range	39–86	39–84	39–86	39–90
Social communication	49.8 (11.1)	49.3 (10.3)	50 (11.5)	47.5 (9.6)
Range	38–90	38–75	38–90	38–86
Social motivation	50.9 (9.2)	50.9 (8.2)	50.9 (9.8)	49.7 (10.5)
Range	37–81	37–65	37–81	37–86
Repetitive behaviors	51.8 (11.1)	53.7 (12.5)*	50.9 (10.4)	48.4 (8)
Range	43–90	43–90	43–90	43–77
Ophthalmological characteristics	n = 60	n = 19	n = 41	n = 46
Visual acuity at far	1.16 (.23)	1.07 (0.27)	1.20 (0.21)	1.19 (.19)
Range		0.32–1.60	0.32–1.60	0.63–1.60
Visual acuity at near	0.97 (.09)	0.92 (0.16)	0.99 (0.04)	1.0 (0.10)
Range		0.40–1.00	0.30–1.00	0.50–1.00
Low contrast visual acuity	0.54 (0.17)	0.47 (0.14)	0.58 (0.17)	0.57 (0.12)
Range		0.05–0.63	0.10–0.80	0.25–1.00

Descriptive statistics for the whole preterm cohort (LOVIS) and the two subgroups VPT and EPT, and the full-term controls. All data is shown as m (sd) unless otherwise specified. Part A: neonatal characteristics. The whole cohort, and sub groups (EPT and VPT) are individually compared with the full-term group with *t*-test for dimensional and χ^2^ for categorical variables. Part B: Not all included children had data for all assessments and group size varies. Group differences were compared using one-way ANOVAs. All statistically significant differences are denoted ^*^p < 0.05 and refers comparisons between the preterm groups with the full-term group. Bonferroni was used as post-hoc test where applicable

#### Biological motion interpretation accuracy in low vs high noise

As seen in Fig. 2, interpretation of biological motion was more challenging in high than in low noise. Within-group differences between these conditions were analysed using the Wilcoxon signed-rank test. The results demonstrated no difference within the EPT group, *Z* = − 1.34, *p* = 0.180, *r* = 0.27 indicating no effect on performance by the higher noise level. The VPT group performed better in low noise (Mdn = 2) as compared to the high noise (Mdn = 1) *Z* = − 4.06 *p* < 0.001, *r* = 0.55, indicating a large effect. Also, the full-term group performed better in the low noise (Mdn = 2), than the high noise (Mdn = 1), *Z* = − 2.35, *p* = 0.019, *r* = 0.33, indicating a medium effect.

#### Group differences in autistic traits

**Fig. 3:**
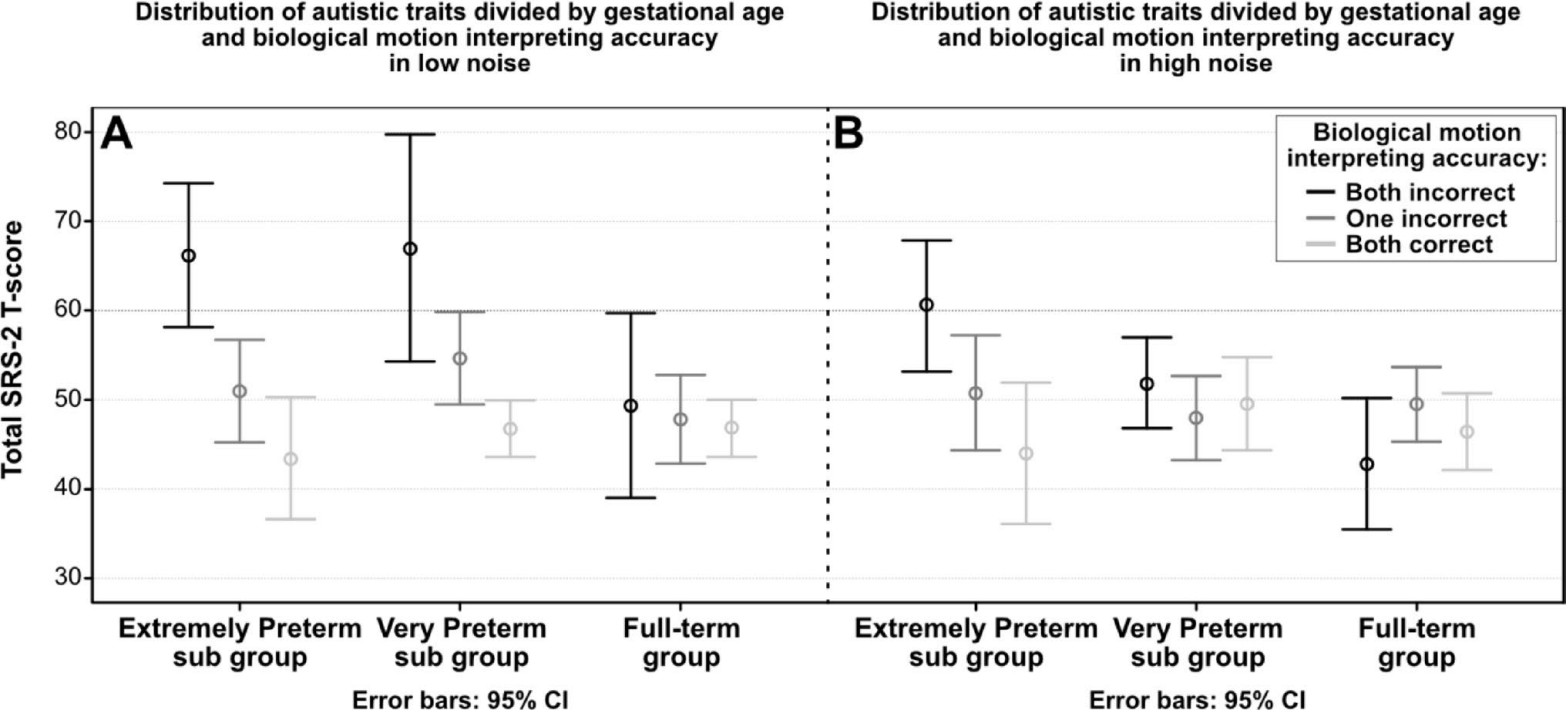
Line diagram distribution of total Social responsiveness Scale-2 Total scores where higher scores indicate more autistic traits. Groups are divided by gestational age groups on the x-axis and the BM interpreting accuracy in low (panel **A**) and high (panel **B**) noise are presented as black- both incorrect interpretations, grey—one incorrect and light grey—both correct for each gestational age group

One-way ANOVAs evaluated the relation between preterm-status (EPT, VPT and full-term) and SRS-2 total score (Fig. 3) and subscales scores. The means and standard deviations are presented in Table 1. Mean difference between groups was statistically significant only for the Restrictive Repetitive Behaviors scores, *F*(2, 113) = 4.83, *p* = 0.010, η^2^ = 0.079, indicating a medium effect. Bonferroni post hoc analysis revealed that only the EPT group (M = 53.7, SD = 12.5) had significantly higher scores than the full-term group (M = 48.4, SD = 8.0) (*p* = 0.008), indicating more problems with repetitive behaviors.

### Background characteristics in relation to biological motion interpretation accuracy and autistic traits

To the primary aim, we first examined associations between neonatal characteristics, ophthalmological characteristics and general intelligence in relation to autistic traits using univariate regressions. These results are presented in Supplementary Table 1. Ophthalmological characteristics showed no associations with BM interpretation accuracy nor autistic traits in any group. *Neonatal characteristics* in the EPT group showed that IVH was associated with more autistic traits. Lower gestational age and not receiving antenatal steroids were also associated with more autistic traits. In the VPT group there were few associations between neonatal characteristics and autistic traits. *General intelligence* was used as a dependent variable with BM interpretation in low and high noise as independent variables, respectively. In the EPT group, lower general intelligence was associated with poorer BM interpretation accuracy in low *F*(1, 22) = 5.89, *p* = 0.024, R^2^ = 0.22 indicating a medium effect, as well as high noise *F*(1, 22) = 4.93, *p* = 0.037, R^2^ = 0.19, indicating a medium effect. In the VPT group, lower general intelligence was associated with poorer BM interpretation accuracy in low noise *F*(1, 47) = 7.29, *p* = 0.010, R^2^ = 0.13, indicating a small effect. Lower intelligence was associated with more autistic traits for some scales in both the EPT and VPT group (supplementary Table 1). There were no significant associations within the full-term group. Significant univariate associations between background factors and BM interpretation accuracy, as well as autistic traits, were later used as covariates in multivariate regressions for the preterm groups.

### Biological motion interpretation accuracy and autistic traits

Univariate and multivariate regression models investigated the relation between BM interpretation accuracy and autistic traits. Table 2 shows regression coefficients for BM interpretation (low/high noise) and autistic trait domains (SRS-2 Total Score, Social Awareness, Social Cognition, Social Communication, Social Motivation, and Repetitive Behaviors), along with variable-specific adjustments with neonatal characteristics and general intelligence. All significant coefficients were negative, indicating that poorer BM interpretation was associated with more autistic traits. In the low noise condition for the EPT group, Social Awareness showed the strongest and most persistent association with BM interpretation accuracy, remaining significant after adjustments. The Total SRS-2 score, Social Cognition, Social Communication and Repetitive Behaviors had strong initial associations that weakened post-adjustment, while Social Motivation were not adjusted due to lack of univariate associations. In high noise, associations were generally weaker, with adjustments having a greater impact. In the VPT group, no adjustments were made due to lack of univariate associations.

**Table 2:** Summary of unadjusted univariate and adjusted multivariate linear regressions for the EPT and VPT subgroups. The control group is not presented as no relations were found

		SRS-2 total score		Social awareness		Social cognition		Social communication		Social motivation	Repetitive behaviours	
		Unadju	Adju^a^	Unadju	Adju^b^	Unadju	Adju^a^	Unadju	Adju^a^	Unadju	Unadju	Adju^a^
		(β)	(β)	(β)	(β)	(β)	(β)	(β)	(β)	(β)	(β)	(β)
EPT group												
Biological motion interpretation accuracy	Low noise	− 0.72***	− 0.53**	− 0.78***	− 0.75***	− 0.65***	− 0.40*	− 0.66***	− 0.47*	− 0.49**	− 0.63***	− 0.37*
	High noise	− 0.57**	− 0.35	− 0.61**	− 0.56**	− 0.58**	− 0.33	− 0.51*	− 0.30	− 0.38	− 0.44*	0.16
VPT group												
Biological motion interpretation accuracy	Low noise	− 0.44**		− 0.37*		− 0.42**		− 0.44**		− 0.40**	− 0.38*	
	High noise	− 0.09		− 0.06		− 0.11		− 0.06		− 0.05	− 0.15	

Unadjusted univariate linear regressions and adjusted multivariate linear regressions presented as β. Dependent variable was the scales from SRS-2 and independent variable are BM interpretation accuracy in different levels of noise. Results are presented for the EPT and VPT sub-groups separately. Please note that depending on univariate associations, different covariates were entered as predictors depending on the group. All statistically significant coefficients are denoted ***p < .001, **p < .01, *p < .05

Variables used in the adjusted models depending on group:

EPT group:

^a^IVH grade 3–4, Full-scale IQ

^b^IVH grade 3–4

VPT group: No adjustment made

Biological motion interpretation accuracy in the VPT group was more weakly associated with autistic traits. High-noise associations were very weak and non-significant across all domains. No significant association between BM interpretation accuracy in either noise level and autistic traits were found for the full-term group.

## Discussion

This study found that poorer BM interpretation accuracy was related to autistic traits in EPT and VPT children. Specifically, issues with social awareness were linked to the BM interpretation accuracy, even when adjusting for neonatal factors, and concurrent ophthalmological factors as well as general intelligence scores. Children born EPT generally had poorer BM interpretation accuracy compared with children born VPT and full-term. Interpretation accuracy was reduced by increased visual noise in the VPT and full-term group. Taken together, the findings suggested that perceptual difficulties in interpretation of biological motion may be of importance for screening and understanding social reciprocity problems for children born EPT and VPT, and that even a condensed assessment might be enough to capture relevant information.

### Biological motion interpretation

When investigating individuals older than five years, BM stimuli are embedded in randomly moving visual noise consisting of moving dots with the same visual and motion properties as the point-light figure dots to introduce visual ambiguity and performance variability (Cutting et al. 1988; Pavlova et al. 2001). This study investigated differences in BM interpretation using a condensed assessment of two noise levels to explore its potential clinical usability. The assessments used an ordinal scale and were presented on an ordinary computer screen in under a minute. Children born very preterm have shown reduced sensitivity to BM in noise and difficulties in BM interpretation compared to full-term peers where noise levels used in this study were in concurrence with ceiling and floor effects observed in previous studies (Taylor et al. 2009; Williamson et al. 2015). We acknowledge that with only two levels of noise, we cannot fully assess potential ceiling or floor effects in this sample. However, the selected noise levels were sufficient to identify a point at which biological motion interpretation became challenging for children born EPT and VPT, as well as for FT controls, capturing meaningful differences in task difficulty without producing extreme floor or ceiling performance. The assessment’s brevity and simplicity in administration and interpretation suggested potential clinical utility for screening BM interpretation.

The findings showed that the EPT group had difficulties in the low noise condition compared to VPT and full-term peers and faced similar challenges in BM interpretation across both low and high noise conditions. Increasing the noise was anticipated to reduce BM interpretation accuracy (Hadad et al. 2011; Pavlova et al. 2001; Williamson et al. 2015). Within-group differences in our study revealed that the EPT group performed equally poorly in both noise levels, whereas the full-term and VPT groups, particularly the latter, had more trouble with interpretation of BM in the high noise condition compared to the low noise condition. The ordinal scale and brevity of the BM assessment may account for lack of further effects. The accuracy in BM interpretation was also associated with general intelligence for both preterm groups. This suggested that socio-cognitive aspects were also important when interpreting the motion of others.

### Biological motion interpretation, dorsal stream vulnerability and ophthalmological factors

Motion processing depends primarily on the dorsal visual stream, which is responsible for detecting motion-related spatial information such as direction, trajectory, and speed. (Atkinson & Braddick 2007; Braddick et al. 2003; Goodale 2013). Dorsal stream functions, such as visuomotor integration have been related to visual acuity and contrast sensitivity in children born very preterm, suggesting that the quality of visual input may influence the development of dorsally related functions (Tu et al. 2024). Our study specifically investigated the relationship between ophthalmological factors and BM interpretation accuracy in children born preterm, but found no significant associations. This is consistent with our previous research on BM detection sensitivity in noise, which also showed no associations to ophthalmological factors (Johansson et al. 2023). Similar findings have been reported in other studies, suggesting that BM processing integrity is independent of ophthalmological function (Taylor et al. 2009). Additionally, children treated for congenital cataracts exhibited unimpaired BM sensitivity following vision-restoring treatment (Hadad et al. 2012). Most studies of BM processing in preterm born populations have used visual acuity as an inclusion criteria rather than a covariate (Pavlova et al. 2003; Williamson et al. 2015). The participants were assessed with normal or corrected to normal visual acuity and which ranged between 0.32 to 1.25 Snellen decimal acuity. As BM was assessed with white dots on black background we also investigated contrast sensitivity on a continuous scale as a potential covariate, whereas we found no relation between low contrast visual acuity and BM interpretation accuracy. Concerning the ophthalmological examination, 18 children in the preterm groups did not participate which limited the findings in this area. These 18 did not differ from the remaining children in the preterm group in Full-scale IQ nor SRS-2 Total scores.

### Biological motion interpretation and autistic traits

Humans are finely tuned to quickly perceive biological motion and use it to infer emotional states and intentions, enabling fluent social interactions (Miller and Saygin 2013; Pavlova 2012). Biological motion processing has been extensively investigated in individuals with ASD and is believed to be closely associated with atypical social and emotional development (Federici et al. 2020). Although basic perceptual abilities related to BM appear to be preserved in ASD, deficits are frequently observed in higher-level interpretation of socially relevant motion cues (Atkinson 2009; Federici et al. 2020). That is, while individuals with ASD may accurately detect BM, their ability to infer goal-directed behavior or social intent from such motion was impaired in relation to neurotypical peers. In neurotypical individuals, the perception and understanding of BM is thought to occur largely automatically and without conscious effort. In contrast, individuals with ASD may rely on compensatory strategies that involve higher-order cognitive functions and general intellectual abilities (Foglia et al. 2022). The bulk of literature focuses on BM processing in ASD, and fewer have concentrated on less pervasive social functioning difficulties in terms of autistic traits. The few previous studies on BM processing in children born preterm have shown deficits in both basic and higher-level interpretation of BM, with no relation to general intelligence (Pavlova et al. 2003; Taylor et al. 2009; Williamson et al. 2015). The BMs in the current study were presented for only one gait-cycle of the movement to better reflect the split-second nature of social interaction in everyday life (Pavlova 2012; Ritchie et al. 2015). It is also possible that it was the motion information conveyed in this time that was of essence in relation to autistic traits as social interaction requires a real-time flow of information and response.

In the LOVIS cohort, the 12-year follow-up revealed an ASD prevalence of 6%, with an additional 8% of children exhibiting severe autistic traits (Johansson et al. 2023; Kochukhova et al. 2022). In this study, we investigated the full spectrum of autistic traits. For the EPT children, BM interpretation accuracy was strongly related to autistic traits, particularly social awareness and motivation for social interaction. These association models showed only weak influences from neonatal and cognitive factors, suggesting that difficulties in BM interpretation might contribute to challenges in recognizing social cues and engaging socially. That means that the perceptual component remained strong even when controlling for general intelligence. Social understanding, communication, and repetitive behaviors were also linked to BM interpretation, though these associations weakened after adjusting for neonatal and cognitive factors. Possibly, cognitive abilities may serve as compensatory mechanisms, helping individuals navigate social interactions despite underlying perceptual difficulties (Laverty et al. 2021; Williamson and Jakobson 2014). Repetitive behavior scores were more strongly associated with neonatal factors, such as low birth weight, IVH, and lack of antenatal steroids, indicating that neonatal characteristics had a greater impact on repetitive behaviors than BM interpretation accuracy. General intelligence was also related to repetitive behaviors as also seen in groups with ASD (Bishop et al. 2006).

The VPT group showed a similar pattern as the EPT group where BM interpretation accuracy linked to autistic traits, though less strongly. This was mainly due to fewer children in the VPT group having poor BM interpretation accuracy in the low noise condition. Neonatal and cognitive factors had a smaller influence on these associations. In contrast, BM interpretation was not associated with autistic traits in the full-term group. The BM task used in this study focused solely on interpretation of walking direction rather than complex emotions or activities, which may have limited the challenge for full-term children where perceptual difficulties and autistic traits were less expected (Carter and Pelphrey 2006; Federici et al. 2020).

In sum, these findings highlighted the importance of BM interpretation in relation to autistic traits in preterm populations. Children born EPT and VPT with the greatest difficulty with BM interpretation in low noise also exhibited the highest levels of autistic traits, often within the clinical range of the SRS-2 (Constantino, 2012). General intelligence was associated with BM interpretation accuracy, and autistic traits, but the relation between the latter remained after adjusting for IQ. While preterm-born children frequently face visual perception challenges, these could relate to their ability to interpret others' movements and navigate social interactions.

This exploratory study suggested that a condensed assessment with two levels of noise was sufficient to detect associations with autistic traits, and that brief evaluations could capture relevant information in clinical settings. The findings are correlational in nature, and could be interpreted as either that the lower performance in BM interpretation is related to a social disinterest going hand in hand with autistic traits, or that lower BM interpretation accuracy is related to difficulties to obtain information necessary for higher-order social cognition. This BM assessment could serve as a preliminary screening measure before administering more extensive questionnaires and conducting further assessments. Future research should examine its applicability across different age groups and noise levels. The results also highlight that biological motion appears to be relevant for understanding autistic traits in children born very preterm, and that this can be assessed with a relatively simple procedure. Also, longitudinal studies might reveal additional clinical uses.

### Methodological discussion

The strengths of this study included the evaluation of prospectively collected neonatal characteristics, ophthalmological characteristics and general intelligence as covariates when investigating the ability to interpret BM. The relatively small sample sizes and the use of an ordinal scale with few steps for BM interpretation assessment may have impacted statistical power of the results. Further, it should be considered that this administration of only one type of movement from one point of view might have missed even more sensitive aspects of biological motion, such as variation in kinematics, coordination, timing, direction, intention and identity of the moving agent (Federici et al. 2020; Foglia et al. 2022; Pavlova 2012; Taylor et al. 2009; Williamson et al. 2015). This BM administration failed to show suggested group differences for the VPT group and highlighted only the more immature EPT group. However, for the primary aim, we found strong relations between BM interpretation and autistic traits in the preterm groups. The use of BM stimuli may be valuable as a screening for autistic traits in children born very preterm, but might also be further refined by varying other parameters in the stimuli.

### Conclusion

This study supported that difficulties in biological motion interpretation related to autistic traits in children born preterm and demonstrated that a brief assessment could yield clinically relevant knowledge. The findings emphasized motion sensitivity relating to social perception and highlighted the importance for screening of social perception in this population. Ophthalmological variables did not relate to BM interpretation accuracy, indicating that difficulties were unlikely to be explained by reduced visual acuity. This research pinpoints specific areas of concern for everyday functioning in children born very preterm, possibly of importance in interventions or facilitating accommodations.

## Supplementary Information

Below is the link to the electronic supplementary material.

Supplementary Material 1

## Data Availability

The dataset analyzed during the current study are not publicly available due to privacy reasons but are available upon reasonable request to the corresponding author.

## Abbreviations

ASD: Autism spectrum disorder
BM: Biological motion
EPT: Extremely preterm
FT: Full term
GA: Gestational age
IVH: Interventricular hemorrhage
LOVIS: Longitudinal study of visuomotor capacity in very preterm infants
PVL: Periventricular leukomalacia
SRS-2: Social Responsiveness Scale, second edition
VPT: Very preterm
WISC-V: Wechsler Intelligence Scales for Children, fifth edition

## Declarations

### Generative AI and AI-assisted technologies in the writing process

During the preparation of this work the authors used ChatGPT in order to improve readability and language. After using this tool, the authors reviewed and edited the text as needed and takes full responsibility for the content of the publication.
