# Supplementary material for: Perceptual interpretation of biological motion relates to autistic traits in children born very preterm
Source: Exp Brain Res. 2025 Nov 4;243(12):242. doi: 10.1007/s00221-025-07186-6 (PMC12586206; doi:10.1007/s00221-025-07186-6)
Supplement: Supplementary file 1 — Supplementary Material 1 [file 221_2025_7186_MOESM1_ESM.docx]

Supplementary Table 1: Summary of univariate regression analyses between neonatal characteristics, BM interpreting general intelligence and autistic traits.

|  |  | **SRS-2 Total score** | **Social Awareness** | **Social Cognition** | **Social Communication** | **Social motivation** | **Repetitive behaviours** |
| --- | --- | --- | --- | --- | --- | --- | --- |
|  | Group | (β) | (β) | (β) | (β) | (β) | (β) |
| **Gestational age** | EPT | -.34* | -.38* | -.45* | -.30 | -.01 | -.27 |
|  | VPT | -.17 | -.26 | -.19 | -.13 | -.19 | -.14 |
| **Birth weight** | EPT | -.46* | -.32 | -.51* | -.45* | -.11 | -.45* |
|  | VPT | .25 | .19 | .29* | .22 | .17 | .26 |
| **Small for gestational age** | EPT | -.25 | .18 | .21 | .26 | -.07 | .32 |
|  | VPT | -.06 | -.01 | -.10 | -.05 | .04 | -.13 |
| **Bronchopulmonary dysplasia** | EPT | .01 | -.05 | .04 | .04 | -.39 | -.17 |
|  | VPT | .05 | .07 | .11 | .08 | -.01 | -.05 |
| **Persistent ductus arteriosus** | EPT | -.05 | -.25 | .07 | -.01 | -.02 | -.05 |
|  | VPT | .07 | .13 | .07 | .11 | .02 | -.02 |
| **Intraventricular  haemorrhage ≥ stage 3** | EPT | .53** | .55** | .47* | .57** | .11 | .51** |
|  | VPT | .11 | .04 | .08 | .18 | .02 | .07 |
| **Periventricular leukomalacia** | EPT |  |  |  |  |  |  |
|  | VPT | -.01 | .10 | -.01 | .01 | -.17 | -.01 |
| **Antenatal steroids  (yes/no)** | EPT | -.34* | -.02 | .16 | -.41* | -.59** | -.37* |
|  | VPT | -.11 | -.01 | -.09 | -.11 | -.24 | -.06 |
| **Retinopathy of prematurity ≥stage 3** | EPT | .26 | .12 | .30 | .29 | .03 | .30 |
|  | VPT |  |  |  |  |  |  |
| **Visual acuity at far** | EPT | -.22 | -.18 | -.37 | -.11 | -.06 | -.20 |
|  | VPT | .05 | -.15 | .02 | .06 | .08 | .19 |
| **Visual acuity at near** | EPT | -.23 | -.18 | -.39 | -.16 | .04 | -.23 |
|  | VPT | -.08 | -.25 | -.04 | -.08 | -.01 | .01 |
| **Low contrast visual acuity** | EPT | -.02 | .02 | -.12 | .05 | .08 | -.13 |
|  | VPT | .16 | .08 | .15 | .19 | .08 | .21 |
| **12 year General intelligence** | EPT | -.59** | -.32 | -.63*** | -.56** | -.26 | -.63*** |
|  | VPT | -.25 | -.35 | -.17 | -.22 | -.17 | -.20 |

Supplementary table 1: Univariate regressions presented as β . All statistically significant associations are denoted ^***^ p < .001, ^**^p < .01, ^*^p < .05. Abbreviations: very preterm group: The whole very preterm cohort n=78, EPT: extremely preterm sub-group n=25, VPT: very preterm sub group n=53, FT. Na: no child in this group had the specific neonatal characteristic.
